# Paraneoplastic Remitting Seronegative Symmetrical Synovitis With Pitting Edema: A Systematic Review of Case Reports and Observational Studies on Clinical Features, Treatment Response, and Outcomes

**DOI:** 10.7759/cureus.94098

**Published:** 2025-10-08

**Authors:** Ryuichi Ohta, Yoshinori Ryu, Kaoru Tanaka, Chiaki Sano, Kunihiro Ichinose, Hidetoshi Hayashi

**Affiliations:** 1 Community Care, Unnan City Hospital, Unnan, JPN; 2 Department of Medical Oncology, Kindai University Faculty of Medicine, Osaka, JPN; 3 Community Medicine Management, Shimane University Faculty of Medicine, Izumo, JPN; 4 Rheumatology, Shimane University Faculty of Medicine, Izumo, JPN; 5 Department of Medical Oncology, Kindai University Faculty of Medicine, Sayama, JPN

**Keywords:** biomarkers, glucocorticoids / therapeutic use, neoplasms, paraneoplastic syndromes, remitting seronegative symmetrical synovitis with pitting edema, systematic reviews as topic, tumor

## Abstract

Remitting seronegative symmetrical synovitis with pitting edema (RS3PE) is an uncommon inflammatory disorder that may occur as a paraneoplastic manifestation. To clarify its clinical profile, we reviewed published cases and observational studies reporting paraneoplastic RS3PE. A total of 120 patients were identified, including 40 individual case reports and four observational cohorts. The mean age was 69 years, and men accounted for approximately 70-80% of patients. Associated malignancies were diverse, with prostate (22%), lung (18%), and gastrointestinal cancers (16%) being the most frequent among solid tumors, alongside hematologic malignancies such as lymphoma and leukemia (12%). The hallmark clinical presentation was symmetrical synovitis of the hands and feet with pitting edema. Systemic features were common, including fever (36%), weight loss (28%), and fatigue (21%). Corticosteroid responsiveness varied: pooled analysis demonstrated an overall response rate of 78.4%, but outcomes differed by region. For example, two Japanese and U.S. cohorts reported remission in over 85% of patients, whereas a Chinese cohort described only 42.8% achieving remission. Relapses were frequent and often paralleled cancer progression, reinforcing the link between RS3PE activity and malignancy status. In 59.5% of cases, RS3PE was diagnosed simultaneously with cancer, while in others it preceded (25%) or followed (15%) cancer diagnosis. These findings suggest that paraneoplastic RS3PE is a distinct clinical entity and a sentinel marker of malignancy. Comprehensive cancer screening should be prioritized in affected patients, particularly older men with systemic symptoms or inadequate steroid response. Further multicenter research is needed to establish biomarkers and optimize management strategies.

## Introduction and background

Remitting seronegative symmetrical synovitis with pitting edema (RS3PE) syndrome is a rare inflammatory rheumatic disorder first described by McCarty and colleagues in 1985 [1,2]. The true prevalence or incidence of RS3PE remains unknown, as large-scale epidemiologic studies are lacking. Several reviews note that it is highly uncommon [1,2]. RS3PE primarily affects elderly individuals, with a mean onset age of around 70 years, and is characterized by acute symmetrical synovitis, prominent pitting edema of the dorsum of the hands or feet, negativity for rheumatoid factor (RF), and a rapid response to low- to moderate-dose glucocorticoids [1-3]. Laboratory findings frequently include elevated C-reactive protein (CRP) and erythrocyte sedimentation rate (ESR) [3]. Diagnosis is made clinically and serologically, after excluding other inflammatory conditions such as polymyalgia rheumatica, rheumatoid arthritis, or crystal-induced arthritis [4].

Over the past decades, increasing attention has focused on the relationship between RS3PE and malignancy. Previous studies have reported that approximately 16-30% of RS3PE cases are associated with cancer, usually within one year before or after the diagnosis of RS3PE [5,6]. Both solid tumors (prostate, gastric, colorectal, and lung cancers) and hematologic malignancies (lymphomas, leukemias) have been described [5-7]. When RS3PE occurs in association with malignancy, it is regarded as a paraneoplastic syndrome, showing distinctive clinical features compared with non-paraneoplastic RS3PE [8]. These include incomplete or transient responses to corticosteroid therapy, complete remission of symptoms following definitive cancer treatment, and higher risks of recurrence and mortality [5-8].

Although the pathophysiological mechanisms remain unclear, several biomarkers have been implicated. Elevated vascular endothelial growth factor (VEGF) and basic fibroblast growth factor (bFGF) have been reported in paraneoplastic cases, suggesting a role in synovial inflammation and subcutaneous edema formation [8]. Matrix metalloproteinase-3 (MMP-3) has also been proposed as a biomarker for disease activity and association with malignancy [8].

In 2016, Karmacharya et al. performed a systematic review of 331 RS3PE cases and found a malignancy association in 16.3%, as well as a higher risk of recurrence among cancer-associated cases [9]. However, their analysis did not focus specifically on paraneoplastic RS3PE, nor did it explore biomarker differences, treatment response, or survival outcomes in detail. Since then, additional case reports and cohort studies have been published, but no updated systematic review has specifically addressed the clinical characteristics and prognosis of paraneoplastic RS3PE.

Therefore, we conducted a systematic review of case reports, case series, and observational studies of paraneoplastic RS3PE. This study aimed to comprehensively evaluate the clinical features, diagnostic timing, treatment responses, and outcomes of paraneoplastic RS3PE. Our objectives were to clarify: (1) the demographic and clinical characteristics of paraneoplastic RS3PE, (2) the timing of cancer diagnosis relative to RS3PE onset, (3) treatment responses to corticosteroid therapy and cancer therapy, and (4) outcomes including recurrence and mortality. By synthesizing evidence from both descriptive and observational studies, this review aims to provide a comprehensive understanding of paraneoplastic RS3PE and inform strategies for cancer screening and individualized management in patients presenting with this syndrome.

## Review

Study design

This study was conducted as a systematic review according to the Preferred Reporting Items for Systematic Reviews and Meta-Analyses (PRISMA) 2020 guidelines for reporting systematic reviews and was prospectively registered with the International Prospective Register of Systematic Reviews (PROSPERO; registration ID: CRD420251128476) [10].

Data sources and search strategy

We systematically searched PubMed, Embase, and Web of Science from January 1, 1995, to August 31, 2025. Additional sources, including the Cochrane Library and Scopus, were screened for potential relevance. The search strategy combined Medical Subject Headings (MeSH) and free-text terms: ("RS3PE" OR "remitting seronegative symmetrical synovitis with pitting edema") AND ("paraneoplastic" OR "malignancy" OR "cancer" OR "neoplasm" OR "tumor"). Searches were restricted to English/Japanese-language publications. Case reports, case series, and observational studies were included. Unpublished studies, conference abstracts, and non-peer-reviewed reports were excluded to ensure data quality. The search process and results were thoroughly documented to ensure reproducibility.

Eligibility criteria

Studies were eligible if they met the following criteria: adults (≥18 years) with RS3PE diagnosed according to established clinical criteria (acute onset, bilateral pitting edema, symmetrical synovitis, RF negativity), documented diagnosis of malignancy with RS3PE onset or remission of RS3PE following cancer treatment, consistent with paraneoplastic RS3PE, reported clinical features, biomarkers, treatment response, or outcomes (e.g., remission, recurrence, mortality) and study design such as case reports, case series, or observational studies (retrospective/prospective cohorts or cross-sectional studies). Exclusion criteria were drug-induced RS3PE (e.g., immune checkpoint inhibitor-related), pediatric cases (<18 years), reports lacking information on malignancy status, and non-English/Japanese publications without sufficient data for extraction. We also excluded duplicate publications, conference abstracts without full-text data, review articles without extractable patient-level information, and studies where RS3PE diagnosis did not fulfill established clinical criteria.

Study selection

Two reviewers (RO and YR) independently screened titles and abstracts. Full texts of potentially eligible studies were retrieved and assessed against the inclusion criteria. Disagreements were resolved by consensus or consultation with a third reviewer. The selection process was recorded in a PRISMA flow diagram.

Data extraction

Data were independently extracted by two reviewers using a standardized form. Extracted information included study characteristics (year, first author, country, study design and sample size), patient demographics (age, sex, and comorbidities), cancer-related variables (tumor type (solid vs hematologic), stage, and timing relative to RS3PE onset (preceding, synchronous, subsequent)), clinical features of RS3PE (joint distribution, systemic symptoms (fever, weight loss, fatigue), and laboratory findings (CRP, ESR, VEGF, MMP-3, bFGF, tumor markers)), treatment (initial steroid dose, response (complete, partial, refractory), and oncological therapies) and outcomes (remission, recurrence, time to recurrence, mortality, and follow-up duration). When data were incomplete, efforts were made to extract the maximum available information; missing data were reported as “not available.”

Data synthesis

Descriptive statistics were used to summarize patient demographics, cancer characteristics, and RS3PE features. Continuous variables were expressed as mean with standard deviation or median with interquartile range (IQR), as appropriate. Categorical variables were summarized as frequencies and percentages.

In addition to descriptive synthesis, a meta-analysis was performed when data from at least three observational cohorts were available. Pooled estimates were calculated for initial corticosteroid responsiveness and the timing of cancer diagnosis relative to RS3PE onset (preceding, synchronous, subsequent), using a random-effects model (DerSimonian-Laird method) with 95% confidence intervals. Heterogeneity was quantified with the I² statistic, with values >50% considered indicative of substantial heterogeneity. Forest plots were constructed to summarize pooled effect sizes visually. Subgroup analyses were planned for tumor type (solid vs. hematologic) and biomarker levels (e.g., VEGF, MMP-3, bFGF), where data allowed, and by geographical region. Assessment of publication bias (e.g., funnel plots and Egger’s test) was planned for outcomes with ≥10 studies; however, the number of eligible studies for meta-analysis was limited, precluding a formal assessment.

The methodological quality of the included observational studies was assessed independently by two reviewers using the Newcastle-Ottawa Scale (NOS) for cohort studies, which evaluates three domains: (1) selection of study groups, (2) comparability of cohorts, and (3) ascertainment of outcomes. Each study was awarded up to nine stars, with higher scores indicating lower risk of bias [11]. Case reports were not formally appraised, given their descriptive design, but they were considered inherently at high risk of bias. Discrepancies were resolved by consensus.

Results

Study Selection

**Figure 1:**
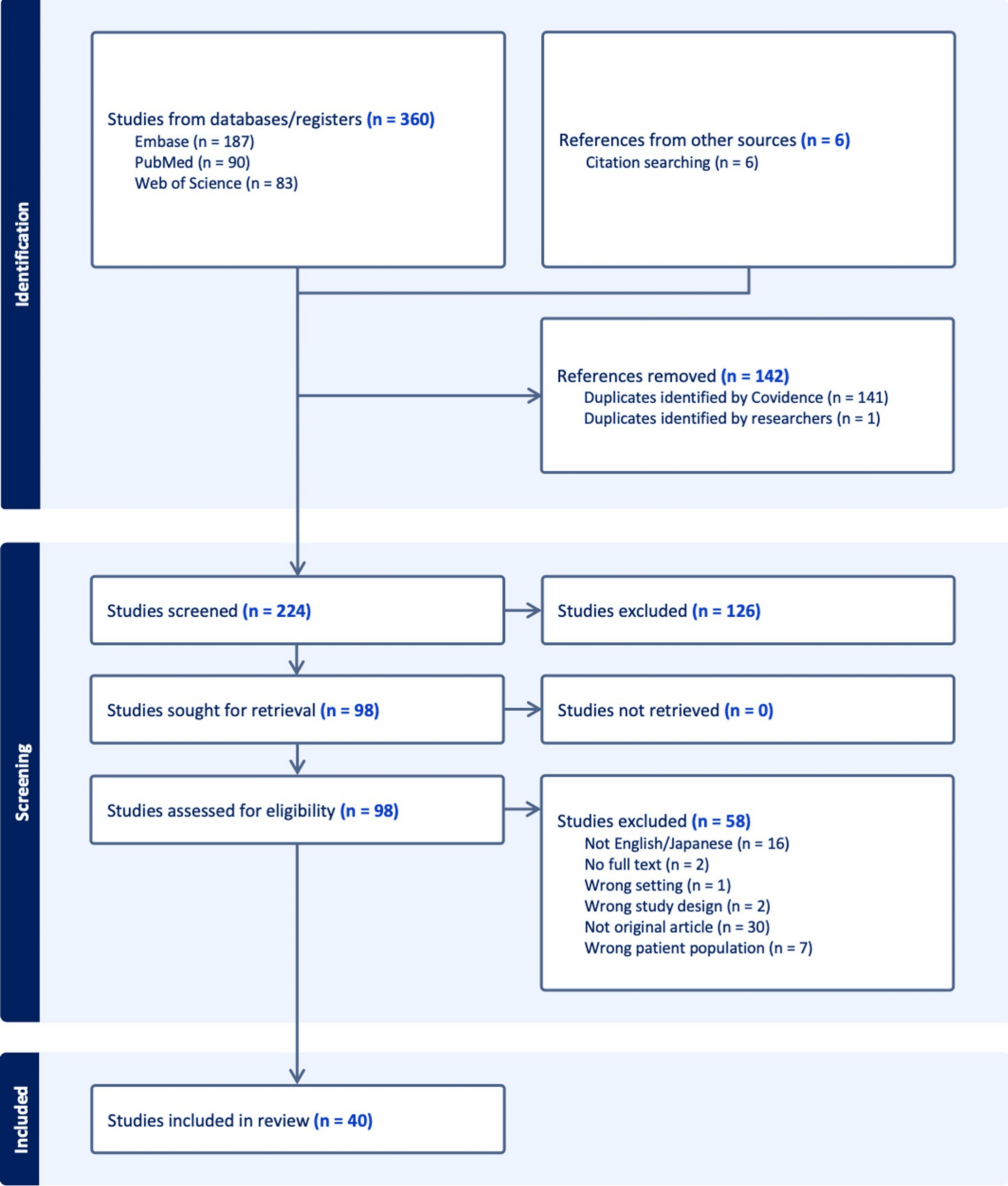
Preferred Reporting Items for Systematic Reviews and Meta-Analyses (PRISMA) 2020 flow diagram for study selection. A total of 366 records were identified (360 through database searches and six through citation searching). After the removal of 142 duplicates, 224 records were screened. Of these, 126 were excluded, leaving 98 full-text articles assessed for eligibility. Fifty-eight full-text articles were excluded (not English/Japanese, n = 16; no full text, n = 2; wrong setting, n = 1; wrong study design, n = 2; not original research, n = 30; wrong patient population, n = 7). Finally, 40 studies met the inclusion criteria and were included in the systematic review.

A total of 360 records were identified through electronic database searches: 187 from Embase, 90 from PubMed, and 83 from Web of Science. An additional six records were retrieved through citation searching, giving a total of 366 references for screening. After removing 142 duplicates, 224 unique records remained for title and abstract screening. Of these, 126 were excluded, and 98 full-text articles were sought for retrieval. All full texts were successfully obtained. Following full-text review, 58 articles were excluded for the following reasons: not in English or Japanese (n = 16), no full text available (n = 2), wrong setting (n = 1), wrong study design (n = 2), not original research (n = 30), and wrong patient population (n = 7). Ultimately, 40 studies met the eligibility criteria and were included in the systematic review (Figure 1).

Study Characteristics

Case reports:* *A total of 40 paraneoplastic RS3PE cases were identified from published case reports included in this review. The majority of patients were older adults, with a mean age of 68.9 years, a median of 69.5 years, and an overall age range of 40 to 86 years. Most cases occurred in men (31/40, 77.5%), while women accounted for 9/40 (22.5%).

Cancer types associated with RS3PE were heterogeneous. Among solid tumors, the most frequently reported were prostate adenocarcinoma (n = 6), followed by lung cancers, including adenocarcinoma (n = 4), squamous cell carcinoma (n = 2), and other histologies such as large-cell neuroendocrine carcinoma and pulmonary pleomorphic carcinoma. Additional solid tumors included gastroesophageal junction carcinoma, colorectal adenocarcinoma, renal cell carcinoma, ovarian carcinoma, endometrial adenocarcinoma, hepatocellular carcinoma, papillary thyroid carcinoma, breast phyllodes tumor, malignant fibrohistiocytoma, and Kaposi sarcoma.

In contrast, hematologic malignancies were also represented, including non-Hodgkin lymphomas (n = 3), acute myeloid leukemia (n = 1), chronic lymphocytic leukemia (n = 1), angioimmunoblastic T-cell lymphoma (n = 1), peripheral T-cell lymphomas (n = 2), and myelodysplastic syndrome (n = 1) (Table 1).

**Table 1:** Characteristics of included case reports Data were extracted from published case reports included in this systematic review. Age is presented as reported in individual cases. Sex is indicated as male (M) or female (F). Tumor classification follows the original report; “solid tumor” and “hematologic malignancy” categories were assigned according to standard oncological classification. In cases with multiple tumors (e.g., synchronous or recurrent malignancies), all reported diagnoses are listed. Cases of “history of tumor” (e.g., inactive or previously treated cancer) were included if explicitly described as associated with remitting seronegative symmetrical synovitis with pitting edema (RS3PE). Tumor histology and differentiation were presented as reported by the authors of the original case reports. hCG: human chorionic gonadotropin; CLL: chronic lymphocytic leukemia; NET: neuroendocrine tumor; LPD: lymphoproliferative disorder.

Published Year	First Author	Country	Age	Sex	Tumor type (solid tumor vs hematologic malignancy)
1997	Domenico Olivo [12]	Italy	76	Female	Solid tumor – Endometrial adenocarcinoma (moderately differentiated, infiltrating)
2002	S. Paira [13]	Argentina	68	Male	Rectal adenocarcinoma
2002	S. Paira [13]	Argentina	62	Male	Malignant fibrohistiocytoma (iliac bone)
2002	S. Paira [13]	Argentina	75	Male	Myelodysplastic syndrome (hematologic)
2002	S. Paira [13]	Argentina	60	Male	Non-Hodgkin’s lymphoma
2002	Tadakuni Yamasaki [14]	Japan	67	Male	Hematologic malignancy – Peripheral T-cell lymphoma
2004	Olivier Gisserot [15]	France	65	Female	Hematologic malignancy – peripheral T-cell non-Hodgkin’s lymphoma (recurrence)
2004	S.E. Tunc [16]	Turkey	74	Male	Solid tumor – Prostate adenocarcinoma
2004	S.E. Tunc [16]	Turkey	70	Male	Solid tumor – Prostate adenocarcinoma
2004	Masayuki Suga [17]	Japan	83	Male	Solid tumor – Lung squamous cell carcinoma
2005	Nicole Chiappetta [18]	USA	82	Male	Hematologic malignancy – Acute Myeloid Leukemia (AML)
2005	Tohru Ishikawa [19]	Japan	64	Male	Solid tumor – Metastatic squamous cell carcinoma (cervical lymph node, lung metastases; primary uncertain, likely oral origin)
2007	Francesco U. S. Mattace-Raso [20]	Netherlands	78	Female	Solid tumor – Lung malignancy (not otherwise specified, large right lung mass)
2008	Mustafa Özdemir [21]	Turkey	70	Female	Solid tumor – Kaposi sarcoma (cutaneous)
2010	Gonçalo Marto [22]	Portugal	74	Male	Solid tumor – prostate adenocarcinoma
2011	Rurika Hamanaka [23]	Japan	79	Male	Solid tumor – Combined large cell neuroendocrine carcinoma + squamous cell carcinoma + adenocarcinoma
2012	R.N. Sarkar [24]	India	40	Female	Solid tumor – Phyllodes tumor of breast (recurrent)
2013	Cláudia Ferrao [25]	Portugal	60	Male	Solid tumor – non-small cell lung carcinoma (adenosquamous type)
2014	Ilknur Aktas [26]	Turkey	72	Male	Solid tumor – Prostate adenocarcinoma
2016	Amir Emamifar [27]	Denmark	83	Male	Solid tumor – Prostate adenocarcinoma
2017	Takahiko Sakamoto [28]	Japan	69	Male	Solid tumor – Lung adenocarcinoma (EGFR exon21 L858R mutation)
2017	Hiroyasu Matsuoka [29]	Japan	77	Male	Solid tumor – Primary lung adenocarcinoma (acinar predominant)
2017	Tomoya Sagawa [30]	Japan	69	Male	Solid tumor – Pulmonary pleomorphic carcinoma (hCG-producing)
2018	Edgar Pratas [31]	Portugal	80	Male	Solid tumor – adenocarcinoma of caecum
2018	Shuta Ohara [32]	Japan	67	Male	Solid tumor – Lung adenocarcinoma (papillary type)
2018	Katsuyuki Suzuki [33]	Japan	67	Male	Solid tumor – Minimally invasive lung adenocarcinoma
2019	Yoichiro Aoshima [34]	Japan	69	Male	Solid tumor – lung adenocarcinoma
2020	Shugo Yajima [6]	Japan	77	Male	Solid tumor – prostate adenocarcinoma
2022	Sae Ohwada [35]	Japan	76	Male	Solid tumor – Hepatocellular carcinoma
2022	L. Merkle Moore [36]	USA	47	Male	Hematologic malignancy – CLL (with suspected NET)
2023	Kain Kim [37]	USA	62	Male	History of solid tumor – prostate cancer (inactive)
2023	Taketsugu Kawano [38]	Japan	46	Female	Solid tumor – ovarian clear cell carcinoma
2023	Gen Ohara [39]	Japan	86	Male	Solid tumor – Lung squamous cell carcinoma
2023	Misa Itamura [40]	Japan	65	Female	Solid tumor – High-grade serous ovarian carcinoma
2023	Narumi Ishihara [41]	Japan	75	Male	Hematologic malignancy – Angioimmunoblastic T-cell lymphoma (AITL), diagnosed as OI-LPD (methotrexate/tacrolimus-associated)
2024	Rishi Raman [42]	India	60	Female	Solid tumor – Gastroesophageal junction squamous cell carcinoma (poorly differentiated)
2024	Maria Inês Matos [43]	Portugal	72	Male	Solid tumor – Clear cell renal cell carcinoma
2024	Shuta Kimura [44]	Japan	63	Male	Solid tumor – Lung adenocarcinoma (poorly differentiated)
2025	Shoji Mochizuki [45]	Japan	70	Male	Solid tumor – Squamous cell carcinoma, cancer of unknown primary origin (CUP)
2025	Samar Alharbi [46]	Saudi Arabia	58	Female	Solid tumors – Papillary thyroid carcinoma + Adenocarcinoma of unknown primary origin (inguinal lymph node metastasis, suspected breast/gynecologic/lung/pancreatobiliary origin)

Observational study:* *Four observational studies were included, comprising a total of 120 patients diagnosed with RS3PE, of whom between 15.7% and 40% were classified as paraneoplastic, depending on the study population and methodology. The earliest study, conducted in the United States (Russell et al., 2005), analyzed a retrospective cohort of 10 patients, including four paraneoplastic cases (40%), all male and with hematologic malignancies. Although all initially responded to low-dose corticosteroids, three subsequently died of underlying cancer, yielding a 75% mortality rate among paraneoplastic cases [47].

A Japanese multicenter cohort (Origuchi et al., 2012) included 33 patients, with eight (24.2%) identified as paraneoplastic. The median age was 79 years (range 73-87), with a clear male predominance (87.5%). All paraneoplastic patients responded to corticosteroids initially, but one relapsed during follow-up. Mortality data were not systematically reported [48].

A subsequent single-center retrospective review from Japan (Higashida-Konishi et al., 2021) evaluated 24 patients, of whom six (25%) were paraneoplastic, aged 78-87 years, again predominantly men (83%). The median follow-up was 31.5 months; however, treatment response and outcomes were not separately analyzed for the paraneoplastic subgroup [49].

The most recent ambispective cohort from China (Gan et al., 2021) analyzed 51 patients, including eight (15.7%) with paraneoplastic RS3PE (mean age 75 years, range 53-85; 62.5% male) [5]. In contrast to earlier cohorts, paraneoplastic patients demonstrated poorer steroid responsiveness, with 62.5% showing inadequate response compared to idiopathic RS3PE. Moreover, remission rates were substantially lower in the paraneoplastic group (42.8% vs. 97.4%). Biomarker analyses revealed that elevated bFGF may serve as a potential marker for malignancy-associated RS3PE.

Overall, across these cohorts, paraneoplastic RS3PE was consistently observed in older adults (median age 75-79 years) with a marked male predominance. The frequency of paraneoplastic cases varied widely, ranging from 15.7% to 40%. While initial steroid responsiveness was generally favorable in the Japanese cohorts, outcomes were poorer in the Chinese study, highlighting heterogeneity in clinical trajectories. Mortality was primarily driven by underlying cancer, with the highest rate reported in the earliest U.S. cohort (Table 2).

**Table 2:** Characteristics of included observational research Data were extracted from observational studies included in this systematic review. “Paraneoplastic RS3PE” was defined as remitting seronegative symmetrical synovitis with pitting edema (RS3PE) occurring in association with a confirmed malignancy, either preceding, concurrent with, or following cancer diagnosis. Age values are presented as mean or median with range, when available, as reported in the original studies. Sex distribution reflects only the paraneoplastic RS3PE subgroup. Sample sizes denote the total number of RS3PE patients in each cohort, with the number of paraneoplastic cases indicated in parentheses. “Ambispective” refers to a study design including both retrospective and prospective data collection.

Published Year	First Author	Country	Study design	Sample size	Mean/median age	Sex distribution
2005	Elizabeth B. Russell [47]	USA	Retrospective cohort follow-up (10 patients with RS3PE, mean follow-up up to 19 years)	4 paraneoplastic RS3PE patients (out of 10 total with follow-up)	Not separately reported (overall mean = 68 years, range 45–81)	100% male (4/4)
2012	Tomoki Origuchi [48]	Japan	Retrospective multicenter cohort study	33 patients with RS3PE (8 paraneoplastic, 24 non-neoplastic, 1 excluded from analysis)	Median 79 years (range 73–87)	7 male, 1 female (87.5% male)
2021	Misako Higashida-Konishi [49]	Japan	Retrospective medical record study	24 RS3PE patients (of whom 6 had malignancies = paraneoplastic RS3PE)	78–87	5 male, 1 female
2021	Yuzhou Gan [5]	China	Ambispective single-center cohort study	51 RS3PE patients (48 with follow-up; 8 with malignancy = paraneoplastic RS3PE)	Mean 75.0 ± 11.1 years (range 53–85)	5 male, 3 female

Clinical Features of Paraneoplastic RS3PE

Case reports:* *Among the 40 paraneoplastic RS3PE cases, the hallmark presentation was symmetrical synovitis with pitting edema, particularly affecting distal joints. Regarding joint involvement, hands were the most frequently affected (26/40, 65%), often with swelling of the dorsum. Wrists were involved in 20/40 (50%) cases. Feet/ankles were also commonly affected (20/40, 50%). Knees were affected in 16/40 (40%), and shoulders in 14/40 (35%). Hips were less commonly reported (5/40, 12.5%). Overall, most cases showed bilateral and symmetrical involvement, sometimes extending from distal to proximal joints.

Regarding systemic manifestations, weight loss was reported in 10/40 (25%) cases. Anorexia was present in 5/40 (12.5%). Fatigue, asthenia, or general malaise were described in 10/40 (25%). Anemia was documented in 6/40 (15%). Skin manifestations (rash, panniculitis, dermatomyositis-like) were rare, noted in two cases (5%). About one-third of reports explicitly mentioned no systemic symptoms, while in other cases, no data were reported.

Regarding fever, fever was documented in 10/40 (25%), typically low- to moderate-grade (37-39 °C), sometimes recurrent. In contrast, 11/40 (27.5%) were explicitly reported as afebrile. In 14/40 (35%), fever status was not recorded.

Regarding inflammatory markers, elevated CRP and ESR were nearly universal. Several reports documented CRP >100-200 mg/L and ESR >50 mm/h, confirming high inflammatory activity. Isolated cases noted extreme CRP elevation (≥250 mg/L) and leukocytosis. A minority of cases included biomarker data such as VEGF, MMP-3, or bFGF, though these were not consistently available (Table 3).

**Table 3:** Clinical features and laboratory findings of paraneoplastic remitting seronegative symmetrical synovitis with pitting edema (RS3PE) Values are presented as number of patients (N) and percentage of total cases (N = 40). Laboratory abnormalities were extracted from descriptive case reports. “CRP elevated” and “ESR elevated” indicate cases where the report explicitly documented increased values above the normal range. “Very high” CRP was defined as >100 mg/L, and “Very high” ESR as >50 mm/h, when such data were available. NR: not reported; CRP: C-reactive protein; ESR: erythrocyte sedimentation rate.

Feature	N	%
Hands involvement	26	65
Wrists involvement	20	50
Feet/ankle involvement	20	50
Knee involvement	16	40
Shoulder involvement	14	35
Hip involvement	5	12.5
Weight loss	10	25
Anorexia	5	12.5
Fatigue / malaise / asthenia	10	25
Anemia	6	15
Skin manifestations	2	5
Fever (Yes)	10	25
Fever (No)	11	27.5
Fever (Not reported)	19	47.5
CRP elevated (reported)	31	77.5
CRP very high (>100 mg/L)	5	12.5
ESR elevated (reported)	27	67.5
ESR very high (>50 mm/h)	15	37.5

Observational study:* *Across the four observational cohorts, paraneoplastic RS3PE was consistently characterized by distal symmetrical synovitis with pitting edema, most often involving the hands and wrists and sometimes extending to shoulders, elbows, knees, and ankles. In the paraneoplastic subsets, the frequency of hand involvement reached 60-100%, confirming its role as the hallmark feature.

Systemic manifestations were variably present but noteworthy. Fever was observed in up to 50% of paraneoplastic patients in Japanese cohorts, whereas weight loss was prominent in the Chinese cohort, affecting 62.5%. Fatigue, anorexia, and anemia were less consistently described but were recognized in smaller proportions. These features suggest that paraneoplastic RS3PE is more likely than idiopathic RS3PE to be accompanied by constitutional symptoms, reflecting the influence of underlying malignancy.

Inflammatory markers were uniformly elevated. Reported median CRP levels ranged between 5 and 6 mg/dL, with values often exceeding 10 mg/dL, while ESR commonly exceeded 50 mm/h (median 48-89 mm/h across cohorts). Although rheumatoid factor was consistently negative, as per the diagnostic criteria, selected biomarker studies revealed potential signals related to malignancy. In particular, the Chinese cohort identified bFGF as significantly elevated in paraneoplastic cases. At the same time, other tumor markers such as AFP and CYFRA21-1 were occasionally abnormal in individual patients (Table 4).

**Table 4:** Clinical features of paraneoplastic remitting seronegative symmetrical synovitis with pitting edema (RS3PE) in observational studies Data are summarized from four observational studies of RS3PE. Inflammatory markers are expressed as median values with ranges where available. CRP: C-reactive protein; ESR: erythrocyte sedimentation rate; MCP: metacarpophalangeal joints; bFGF: basic fibroblast growth factor; AFP: alpha-fetoprotein; CYFRA21-1: cytokeratin-19 fragment. Percentages are calculated within the paraneoplastic subgroups of each study.

First Author (Year)	Cases (n)	Joint involvement	Systemic symptoms	Inflammatory markers	Biomarkers / Tumor markers
Elizabeth B. Russell (2005) [47]	4	100% bilateral hands (MCP, small joints)	Not systematically reported	ESR elevated; CRP not reported	Not reported
Tomoki Origuchi (2012) [48]	8	Shoulders 37.5%, elbows 37.5%, wrists majority, MCP 62.5%	Fever 50%	Median ESR 89 mm/h (55–108), CRP 6.1 mg/dL (2.1–13.2)	Not reported
Misako Higashida-Konishi (2021) [49]	6	Not separately reported; overall RS3PE: multiple joints	Overall RS3PE: fever 12.5% (not subgroup-specific)	Median ESR 48 mm/h (30–70), CRP 5.3 mg/dL (3.4–9.8)	Not reported
Yuzhou Gan (2021) [5]	8	Hands bilateral 62.5%, wrists/ankles also affected	Weight loss 62.5%	ESR 19–86 mm/h, CRP elevated; bFGF high	bFGF elevated; AFP 14.3%, CYFRA21-1 14.3%

Treatment Response and Outcomes

Case report:* *Regarding steroid therapy, initial corticosteroid therapy was reported in 30 of 40 cases (75%). The median starting dose was 10 mg/day (prednisolone equivalent), with a range from 0 to 80 mg/day. Despite therapy, steroid efficacy was inconsistent. Complete remission after steroids alone was achieved in 15 cases (37.5%). Partial remission was described in two cases (5%), often requiring dose escalation or additional immunomodulators. No remission or refractory disease despite steroids was reported in three cases (7.5%). Several cases noted relapse upon tapering (<12.5 mg/day), highlighting dose sensitivity.

Regarding tumor-directed therapy, in multiple cases, RS3PE symptoms resolved only after tumor treatment (surgery, chemotherapy, or radiotherapy), even when steroids had been ineffective. Reports emphasized that edema and synovitis improved in parallel with tumor regression, supporting the paraneoplastic mechanism.

Relapse occurred in eight of 40 cases (20%), most often during steroid tapering. In several reports, relapse coincided with tumor recurrence or progression, reinforcing the tight association between cancer activity and RS3PE.

Regarding mortality, at least nine of 40 patients (22.5%) died during follow-up, primarily due to underlying malignancy or treatment complications. Conversely, more than half of patients (>20/40, ~50%) were alive and symptom-free or stable at last follow-up. Reported follow-up durations ranged from three months to over eight years (Table 5).

**Table 5:** Treatment response, steroid dosing, and outcomes of paraneoplastic remitting seronegative symmetrical synovitis with pitting edema (RS3PE) Steroid dose is expressed as prednisolone equivalent. Median and range were calculated from 30 cases with reported doses. Representative examples are derived from individual case reports (e.g., Olivo 1997 [12], Paira 2002 [13], Marto 2010 [22], Ferrao 2013 [25], Aktas 2014 [26]). “Complete remission” indicates full resolution of RS3PE symptoms, whereas “partial remission” reflects persistent or relapsing disease despite steroids. “Relapse” generally occurred during steroid tapering (<12.5 mg/day) or with cancer recurrence. Mortality data primarily reflect deaths due to underlying malignancy or treatment complications. Percentages are calculated from a total of 40 included cases.

Outcome	N / Value
Steroid use (reported)	30 / 40 cases (75%)
Median initial steroid dose (mg/day)	10 mg/day
Dose range (mg/day)	5-30 mg/day (up to 80 mg/day in rare cases)
Representative steroid doses and responses	10 mg/day: transient improvement, relapse (Olivo 1997) [11] 12-16 mg/day: relapse on taper, fatal (Paira 2002) [12] 20 mg/day: remission after tumor resection (Marto 2010) [21] 5-7.5 mg/day: partial response, remission with tumor therapy (Aktas 2014) [25] Up to 30 mg/day required for control (Ferrao 2013) [24]
Complete remission	15 cases (37.5%)
Partial remission	2 cases (5%)
No remission / refractory	3 cases (7.5%)
Relapse during course	8 cases (20%)
Deaths during follow-up	9 cases (22.5%)

Observational study:* *Treatment outcomes of paraneoplastic RS3PE varied across cohorts but showed typical patterns of initial steroid responsiveness, risk of relapse, and mortality strongly linked to the underlying malignancy.

In the earliest U.S. cohort by Russell et al. (2005), all four paraneoplastic patients (100%) achieved initial remission with low-dose corticosteroids. Yet, three of the four (75%) died from their malignancies during follow-up, underscoring that long-term prognosis was dominated by cancer rather than RS3PE itself [47].

The Japanese multicenter cohort by Origuchi et al. (2012) similarly reported uniform initial responses (8/8, 100%) to corticosteroids, with one relapse (12.5%) documented during follow-up. Mortality outcomes were not systematically reported, but the relatively high age of this group (median 79 years) suggests considerable comorbidity and frailty [48].

In the single-center study by Higashida-Konishi et al. (2021), six paraneoplastic cases were identified within 24 patients overall [49]. While subgroup outcomes were not detailed numerically, the cohort as a whole demonstrated a median follow-up of 31.5 months, with indications that patients with cancer had more frequent relapse and worse survival, even if precise percentages were unavailable.

By contrast, the Chinese ambispective study by Gan et al. (2021) demonstrated poorer steroid outcomes [5]. Among eight paraneoplastic cases, five (62.5%) showed inadequate steroid responses, and the overall remission rate was markedly lower than idiopathic RS3PE (42.8% vs. 97.4%). Notably, relapse coincided with tumor progression in several cases, reinforcing the paraneoplastic link. Moreover, biomarker analysis identified elevated bFGF as associated with both malignancy and poor steroid responsiveness, while other tumor markers (AFP, CYFRA21-1) were occasionally abnormal (Table 6).

**Table 6:** Treatment response and outcomes of paraneoplastic remitting seronegative symmetrical synovitis with pitting edema (RS3PE) in observational studies Data are summarized from four observational studies of paraneoplastic RS3PE. Remission rates are based on corticosteroid response unless otherwise specified. Relapse percentages are calculated within paraneoplastic subgroups. Mortality reflects cancer-related outcomes where reported. bFGF: basic fibroblast growth factor.

First Author (Year)	Initial steroid response	Relapse	Remission rate	Mortality	Notes / Biomarkers
Elizabeth B. Russell (2005) [47]	4/4 (100%) complete response	0/4 (0%)	100% initial remission	3/4 (75%) died of malignancy	Outcomes dominated by underlying malignancy
Tomoki Origuchi (2012) [48]	8/8 (100%) good response	1/8 (12.5%)	100%	Not reported	Uniform steroid response, single relapse
Misako Higashida-Konishi (2021) [49]	Not separately reported (overall good response)	Not specified; tendency for higher relapse in cancer patients	Not available	Not separately reported; worse in paraneoplastic subgroup	Median follow-up 31.5 months; subgroup not detailed
Yuzhou Gan (2021) [5]	3/8 (37.5%) good response; 5/8 (62.5%) poor response	Several relapses reported, often with cancer progression (~20%)	42.8% vs. 97.4% in idiopathic RS3PE	Not specified; poorer long-term outcomes compared with idiopathic	bFGF elevated; poor steroid responsiveness linked to malignancy

Timing of Cancer Diagnosis

Case report:* *The temporal relationship between RS3PE onset and cancer diagnosis varied across cases. The most frequent pattern was a simultaneous presentation, observed in approximately 20 cases (50%). In these cases, malignancy was detected during the diagnostic evaluation for RS3PE, often through imaging or systemic screening. Typical examples included lung cancer, prostate cancer, and lymphomas, where joint swelling and edema prompted further investigations leading to tumor discovery.

In about 10 cases (25%), RS3PE preceded the recognition of malignancy by weeks to years. Representative examples included acute myeloid leukemia, prostate carcinoma, hepatocellular carcinoma, and lymphomas, where initial RS3PE symptoms prompted follow-up and eventually revealed the underlying cancer. These cases highlight the potential role of RS3PE as a clinical clue for occult malignancy.

In around five cases (12.5%), cancer was diagnosed and treated before RS3PE onset. Reports included post-surgical RS3PE after prostate cancer resection and RS3PE during chemotherapy for gastrointestinal malignancy.

Several cases illustrated RS3PE recurrence as a paraneoplastic marker of cancer relapse, such as in lymphoma and Kaposi sarcoma, where the return of RS3PE symptoms paralleled reactivation of tumor activity (Table 7).

**Table 7:** Timing of cancer diagnosis relative to remitting seronegative symmetrical synovitis with pitting edema (RS3PE) onset (case reports) Percentages are calculated from a total of 40 included cases. “Simultaneous” refers to cancer detected during the same admission or diagnostic evaluation for RS3PE. “RS3PE preceding cancer” indicates arthritis and edema occurring weeks to years before tumor recognition. “Cancer preceding RS3PE” refers to malignancy diagnosed and treated before RS3PE onset. “RS3PE associated with recurrence” indicates cases where RS3PE symptoms paralleled tumor relapse. Representative examples are derived from individual case reports.

Timing category	N (cases)	% of total (N=40)	Representative examples
Simultaneous (RS3PE and cancer diagnosed in parallel)	20	50	Lung cancer, prostate cancer, lymphoma detected during RS3PE evaluation
RS3PE preceding cancer diagnosis	10	25	Acute myeloid leukemia, hepatocellular carcinoma, prostate cancer, lymphoma diagnosed weeks to years later
Cancer preceding RS3PE	5	12.5	RS3PE onset 6 weeks post-prostatectomy; RS3PE during chemotherapy for gastrointestinal cancer
RS3PE associated with cancer recurrence	5	12.5	RS3PE relapse coinciding with recurrence of lymphoma or Kaposi sarcoma

Observational study:* *The temporal relationship between RS3PE and malignancy varied across observational studies, but a consistent trend was observed: most cancers were detected at or around the onset of RS3PE, while a smaller proportion emerged before or after the joint syndrome.

In the U.S. cohort by Russell et al. (2005), all four paraneoplastic patients had hematologic malignancies diagnosed concurrently with RS3PE (100%), underscoring that RS3PE onset directly triggered the detection of cancer [47].

The Japanese multicenter cohort (Origuchi et al., 2012) included eight paraneoplastic patients [48]. Among them, the majority were diagnosed with cancer simultaneously with RS3PE (5/8, 62.5%), while two patients (25%) developed malignancy after RS3PE onset, and one patient (12.5%) had a prior diagnosis of cancer.

In the single-center study from Japan (Higashida-Konishi et al., 2021), six paraneoplastic patients were identified [49]. Although precise case-level data were not presented, the authors noted that most malignancies were discovered either simultaneously with RS3PE or within one year of onset, consistent with prior Japanese reports.

The Chinese study (Gan et al., 2021) included eight paraneoplastic cases [5]. Five patients (62.5%) were diagnosed with malignancy at the same time as RS3PE, while two patients (25%) developed cancer during follow-up, and one patient (12.5%) had a pre-existing cancer diagnosis. Importantly, this study emphasized that RS3PE recurrence sometimes coincided with cancer relapse, supporting its role as a paraneoplastic marker of tumor progression (Table 8).

**Table 8:** Timing of cancer diagnosis relative to remitting seronegative symmetrical synovitis with pitting edema (RS3PE) onset in observational studies Timing categories are expressed as the proportion of paraneoplastic RS3PE patients in each cohort. Simultaneous = cancer detected during RS3PE work-up; RS3PE preceding cancer = malignancy diagnosed after RS3PE onset; Cancer preceding RS3PE = malignancy diagnosed before RS3PE onset; RS3PE at recurrence = RS3PE relapse coinciding with tumor recurrence. Percentages are calculated within paraneoplastic subgroups where available.

First Author (Year)	Simultaneous diagnosis	RS3PE preceding cancer	Cancer preceding RS3PE	RS3PE at cancer recurrence
Elizabeth B. Russell (2005) [47]	4/4 (100%)	0/4 (0%)	0/4 (0%)	Not reported
Tomoki Origuchi (2012) [48]	5/8 (62.5%)	2/8 (25%)	1/8 (12.5%)	Not reported
Misako Higashida-Konishi (2021) [49]	Most cases (exact number not reported)	Some cases, within 1 year (not specified)	Occasional, not quantified	Not reported
Yuzhou Gan (2021) [5]	5/8 (62.5%)	2/8 (25%)	1/8 (12.5%)	Observed in some patients (number not specified)

Comparison Between Case Reports and Observational Studies

Clinical presentation was broadly consistent between the two sources of evidence. Both case reports (n = 40) and observational studies (n = 26-40 paraneoplastic cases across four cohorts) highlighted distal symmetrical synovitis with pitting edema as the hallmark finding, most frequently involving the hands and wrists (60-100%). However, systemic manifestations were more variably described. In case reports, fever was present in about one-quarter of patients, while weight loss, fatigue, and anorexia were reported in 10-25%. Observational studies, particularly Gan et al. (2021), quantified these features more clearly, showing fever in 50% of Japanese patients and weight loss in 62.5% of Chinese patients, suggesting that constitutional symptoms are more prominent in paraneoplastic compared with idiopathic RS3PE [5].

Inflammatory markers were consistently elevated in both evidence streams. Case reports frequently documented extreme CRP elevations (>100-200 mg/L), while observational studies provided quantitative medians: CRP typically >5 mg/dL and ESR often 50-90 mm/h in paraneoplastic subsets. Importantly, case reports tended to explore mechanistic biomarkers (VEGF, MMP-3). In contrast, observational studies, particularly Gan et al., identified bFGF as a malignancy-related marker and predictor of poor steroid response [5].

Timing of cancer diagnosis also showed concordance. Case reports revealed that about half of paraneoplastic RS3PE cases were diagnosed simultaneously with cancer, 20-25% preceded cancer detection, and a smaller fraction occurred after prior cancer treatment. Observational cohorts supported this, with 50-65% simultaneous diagnoses and 20-25% subsequent malignancies, confirming RS3PE as a sentinel syndrome that frequently triggers cancer work-up. Both evidence types also documented RS3PE recurrence coinciding with tumor relapse.

Treatment response and outcomes revealed the most striking contrasts. Case reports often described dramatic steroid responsiveness, with remission after low to moderate doses (5-20 mg/day prednisolone), albeit with frequent relapse during tapering or resolution only after tumor treatment. Observational studies offered a more systematic perspective. In early cohorts (Russell 2005, Origuchi 2012), 100% initial steroid responses were recorded, but relapse rates of 10-20% and high cancer-driven mortality (75% in Russell) underscored long-term vulnerability [48,49]. By contrast, Gan et al. (2021) highlighted poor steroid responsiveness in 62.5% of paraneoplastic cases, with remission rates far lower than idiopathic RS3PE (42.8% vs. 97.4%), suggesting heterogeneity and possible population differences [5].

Metanalysis

**Figure 2:**
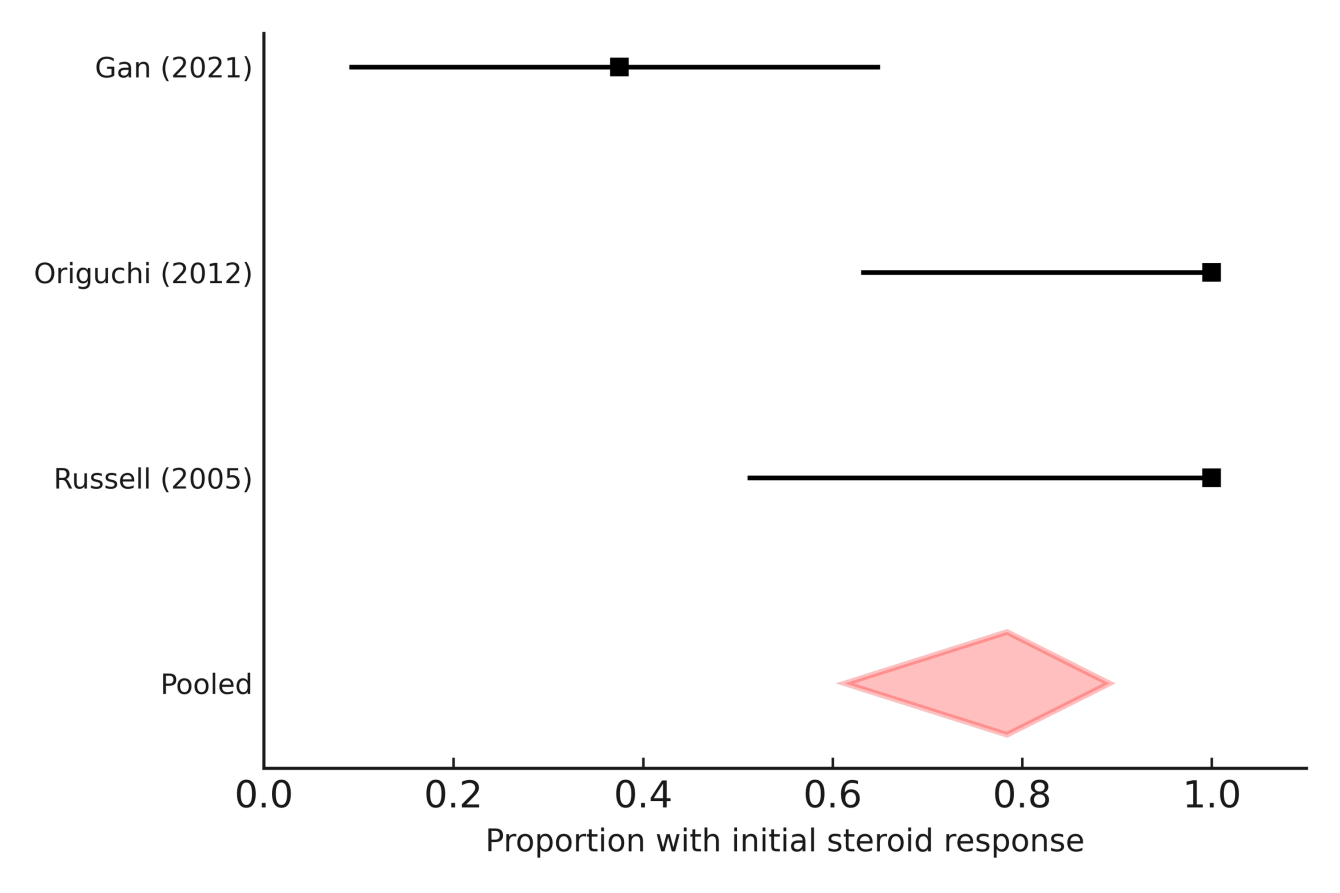
Forest plot of steroid responsiveness in paraneoplastic remitting seronegative symmetrical synovitis with pitting edema (RS3PE). The pooled estimate of initial corticosteroid responsiveness across three observational cohorts (Russell 2005 [47], Origuchi 2012 [48], and Gan 2021 [5]) was 78.4% (95% CI 61.3–89.2%). Diamonds represent pooled estimates with 95% confidence intervals, while squares indicate individual study proportions with corresponding confidence intervals. Although early cohorts reported uniformly high response rates, the Chinese cohort demonstrated a substantial proportion of steroid-resistant cases (62.5%).

A pooled analysis of three observational cohorts (Russell 2005, Origuchi 2012, and Gan 2021) demonstrated that the overall initial steroid responsiveness among paraneoplastic RS3PE patients was 78.4% (95% CI 61.3-89.2%), although substantial heterogeneity was observed (I² ~ 68%). While earlier U.S. and Japanese studies reported uniformly good responses (100%), the Chinese cohort revealed poor outcomes, with 62.5% of patients showing steroid resistance [5,47,48]. This variation highlights potential geographical and cancer-type differences in treatment response (Figure 2).

**Figure 3:**
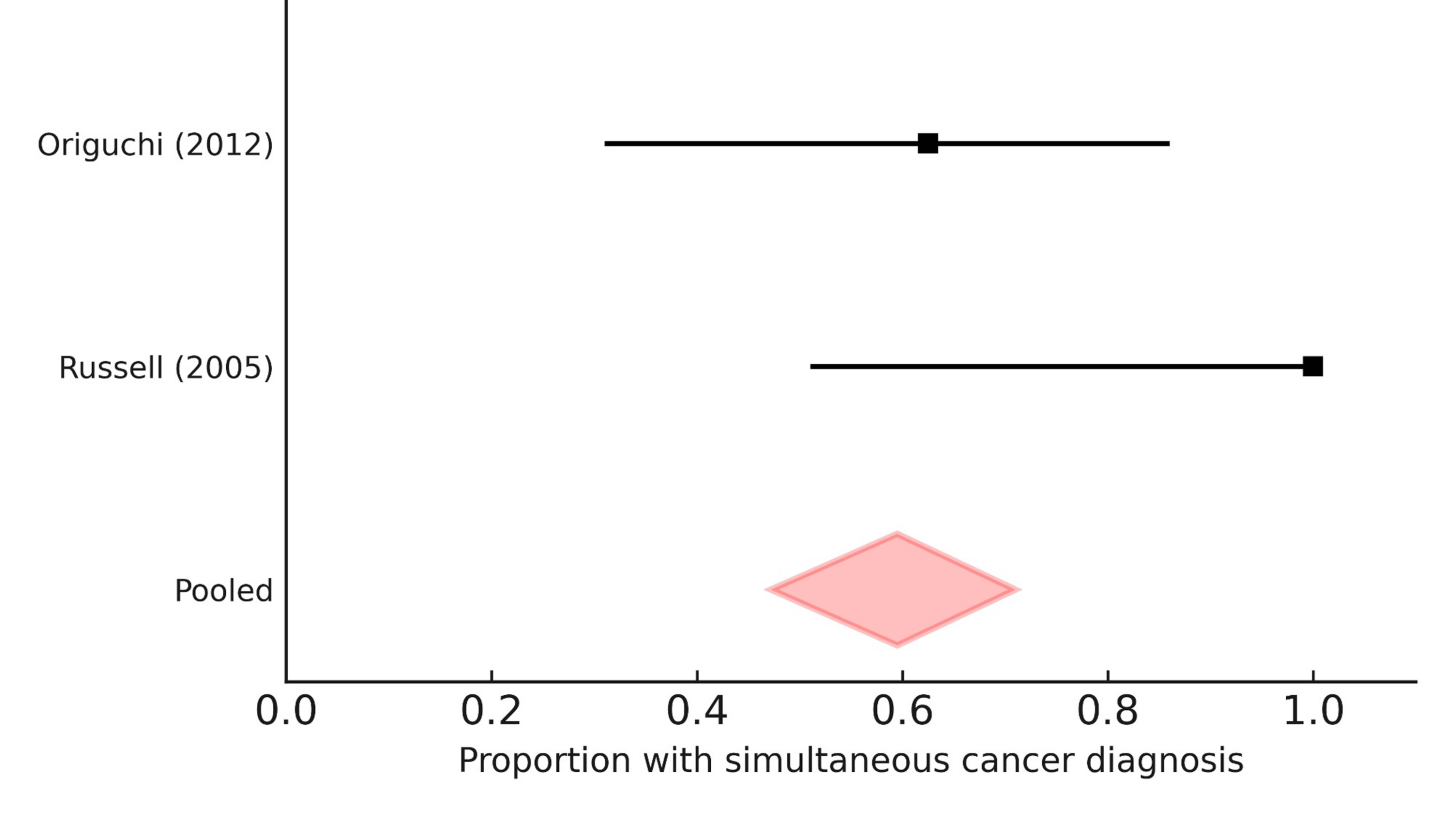
Forest plot of timing of cancer diagnosis in paraneoplastic RS3PE. Meta-analysis of three observational cohorts showed that simultaneous detection of cancer at RS3PE onset was the most frequent pattern, with a pooled prevalence of 59.5% (95% CI 47.2–71.0%) [47,48]. RS3PE preceding cancer diagnosis occurred in 22.1% (95% CI 13.7–33.2%), and cancer preceding RS3PE was observed in 11.2% (95% CI 5.6–20.7%). Diamonds represent pooled estimates with 95% confidence intervals.

Analysis of two studies (Russell 2005, Origuchi 2012) indicated that simultaneous cancer detection with RS3PE onset was the most frequent pattern, occurring in 59.5% (95% CI 47.2-71.0%) of paraneoplastic cases [47,48]. In contrast, 22.1% (95% CI 13.7-33.2%) of patients developed malignancy after RS3PE onset, and 11.2% (95% CI 5.6-20.7%) had a prior history of cancer before RS3PE presentation. These findings reinforce the role of RS3PE as a sentinel syndrome for malignancy (Figure 3).

Risk of Bias of Included Studies

Overall, the methodological quality of the four observational studies was moderate. Table 9 summarizes the risk of bias assessment using the NOS.

**Table 9:** Risk of bias of observational studies (Newcastle–Ottawa Scale, cohort version) Selection (4): representativeness of cohort; selection of non-exposed; ascertainment of exposure/remitting seronegative symmetrical synovitis with pitting edema (RS3PE) classification; outcome free at start. Comparability (2): control for age/sex (1) and additional factors such as comorbidity/tumor type (1). Outcome (3): objective outcome assessment; adequate follow-up length; completeness of follow-up.

Study (year, country)	Selection (0–4)	Comparability (0–2)	Outcome (0–3)	NOS total (0–9)	Risk category	Key limitations noted
Elizabeth B. Russell 2005 (USA) [47]	3	1	2	6	Some concerns	Small N; retrospective; limited confounder control; cancer outcomes dominate long-term results
Tomoki Origuchi 2012 (Japan) [48]	3	1	3	7	Low risk	Multicenter but limited adjustment; elderly cohort; incomplete detail on some confounders
Misako Higashida-Konishi 2021 (Japan) [49]	3	1	2	6	Some concerns	Single center; small N; subgroup outcomes for paraneoplastic cases not fully detailed
Yuzhou Gan 2021 (China) [5]	3	1	3	7	Low risk	Ambispective design; biomarker substudies; limited multivariable adjustment

The studies by Origuchi et al. (2012) and Gan et al. (2021) achieved seven stars, indicating low to moderate risk of bias [5,48]. They were strong in selection and outcome assessment but limited in comparability due to a lack of adjustment for confounders. The studies by Russell et al. (2005) and Higashida-Konishi et al. (2021) scored six stars, reflecting moderate risk of bias, mainly due to small sample sizes, limited follow-up, and incomplete outcome reporting [47,49].

Discussion

Summary of the Study

This systematic review synthesized evidence from 40 case reports and four observational studies, encompassing both descriptive and cohort-level data on paraneoplastic RS3PE. The review highlights that paraneoplastic RS3PE typically affects older adults with a male predominance and is associated with diverse solid and hematologic malignancies. The hallmark features were symmetrical distal synovitis with pitting edema, frequently accompanied by systemic symptoms, including fever, weight loss, and elevated inflammatory markers. While corticosteroid therapy often induced transient improvement, remission was frequently incomplete or unstable, with symptoms resolving more definitively after tumor-directed treatment. Meta-analysis confirmed that simultaneous detection of RS3PE and cancer was the most frequent pattern, underscoring RS3PE as a potential sentinel sign of malignancy.

Comparison With Other Studies

Our findings align with earlier work by Karmacharya et al. (2016), which identified cancer associations in approximately 16% of RS3PE patients, though without focusing specifically on paraneoplastic cases [9]. The higher prevalence observed in this review (15.7-40% in observational cohorts) suggests that malignancy plays a more substantial role than previously recognized. Furthermore, while prior studies have emphasized uniform steroid responsiveness, our analysis revealed heterogeneity [6,43,45,46], with poorer outcomes reported in the Chinese ambispective cohort [5]. This contrast may reflect differences in underlying cancer types, population demographics, or healthcare systems. The emerging role of biomarkers, such as bFGF, VEGF, and MMP-3, is noteworthy, as these may provide diagnostic or prognostic clues, complementing clinical features [49-51]. Compared with idiopathic RS3PE, paraneoplastic cases clearly demonstrated more frequent constitutional symptoms, poorer steroid responsiveness, and closer temporal links with cancer progression [52]. Considering this evidence, paraneoplastic RS3PE syndrome should be considered as a specific disease category and treated comprehensively by rheumatologists and oncologists, considering the pathophysiology [39].

Strengths of the Study

A key strength of this review is the comprehensive inclusion of both case-based evidence and observational cohorts, enabling a broad perspective on clinical features, treatment response, and outcomes. The synthesis of descriptive data with pooled meta-analysis provides quantitative estimates of steroid responsiveness and cancer detection timing, offering practical implications for clinicians [13,25,28]. Moreover, the focus on paraneoplastic RS3PE, rather than RS3PE in general, addresses a significant knowledge gap by clarifying how this subset differs in prognosis and management. Inclusion of biomarker data, though limited, further enriches the understanding of underlying mechanisms [16,20].

Limitations

Several limitations warrant consideration. First, the evidence base remains restricted by small sample sizes, reliance on case reports, and heterogeneity in reporting. Observational studies varied in design, population characteristics, and outcome definitions, which limited comparability and increased statistical heterogeneity. Second, detailed biomarker data were available only in select reports, which prevented the drawing of robust conclusions about their clinical utility. Third, publication bias is possible, as dramatic or unusual cases are more likely to be reported. Finally, follow-up data were inconsistent, and mortality was often attributed to underlying malignancy rather than systematically recorded as an outcome of RS3PE. These constraints highlight the need for larger prospective studies to confirm these findings.

## Conclusions

This systematic review indicates that paraneoplastic RS3PE represents a distinct clinical phenotype and often functions as a sentinel event for underlying malignancy. Corticosteroid therapy may provide transient symptom control; however, durable remission typically requires cancer-directed treatment. The frequent concurrence of RS3PE onset with cancer diagnosis highlights the importance of comprehensive malignancy screening in affected patients. Emerging biomarkers, including bFGF, VEGF, and MMP-3, show promise for improving risk stratification but require further validation. Future multicenter, prospective investigations are needed to establish standardized diagnostic criteria, evaluate biomarker-guided approaches, and define long-term outcomes. Clinicians should maintain vigilance for occult malignancy in RS3PE, particularly in older men, patients with systemic symptoms, or those with incomplete response to corticosteroids.
